# Transforming Alzheimer's Care: The Impact of Biomarker Innovations

**DOI:** 10.1002/alz70856_103693

**Published:** 2025-12-25

**Authors:** Paolo Vitali, Nesrine Rahmouni, Yansheng Zheng, Pedro Rosa‐Neto, Marina P Gonçalves, Tevy Chan, Sonia Bélanger, Felix Pageau, Ziad Nasreddine, Marie Christine Le Bourdais, Thomas Tannou, Guy Lacombe

**Affiliations:** ^1^ The McGill University Research Centre for Studies in Aging, Montreal, QC, Canada; ^2^ Douglas Mental Health University Institute, Montreal, QC, Canada; ^3^ McGill University, Montreal, QC, Canada; ^4^ Montreal Neurological Institute, Montreal, QC, Canada; ^5^ Translational Neuroimaging Laboratory, The McGill University Research Centre for Studies in Aging, Montréal, QC, Canada; ^6^ Ministre des Ainés ‐ Ministère de la Santé et des Services sociaux, Quebec, QC, Canada; ^7^ Université Laval, Québec, QC, Canada; ^8^ MoCA Clinic and Institute, Greenfield Park, QC, Canada; ^9^ Alzheimer Society of Montreal, Montreal, QC, Canada; ^10^ CRIUGM, CIUSS Centre‐Sud de l'Ile de Montréal, Montreal, QC, Canada; ^11^ Sherbrooke University, Sherbbrooke, QC, Canada; ^12^ CIUSSS de l'Estrie‐CHUS, Sherbrooke, QC, Canada

## Abstract

Biomarkers of Alzheimer's pathology have undeniably changed the way we diagnose Alzheimer's Disease today. The theoretical, methodological, and technical advances of recent years, which now allow us to identify in vivo the pathophysiological cascade that ultimately leads to dementia, have made it possible to diagnose Alzheimer's Disease accurately and earlier. This has led to an official redefinition of Alzheimer's Disease on a biological basis, no longer centered on the post‐mortem clinico‐pathological correspondence, but on the identification of specific biomarkers of the disease, namely beta‐amyloid protein and phosphorylated tau protein.

The McGill Centre for Studies in Aging (MCSA) in Montreal is internationally recognized as one of the leading research centres in the development and clinical application of biomarkers in neurodegenerative diseases, particularly Alzheimer's Disease. With unique access to sophisticated techniques such as particle analysis by immunodetection assay in cerebrospinal fluid and molecular neuroimaging by Amyloid and Tau PET, the MCSA has biologically characterized hundreds of individuals along the Alzheimer's Disease continuum (preclinical stage, mild cognitive impairment, and dementia). This clinical research activity has not only been highly recognized scientifically but has also improved patient care.

More recently, the emergence of plasma biomarkers is likely to bring about a true revolution in the diagnostic and therapeutic field of Alzheimer's Disease, with the promise of offering accurate, early, and easily accessible screening to the entire aging population through a simple blood test. The advantages of this new biomarker analysis modality, which is less invasive and as effective as cerebrospinal fluid analysis and amyloid PET, are evident.

These innovative practices bring about meaningful changes and positive impacts sought by our task force for our target population. The MCSA, with its unique expertise in the field of biomarkers, aims to improve disease knowledge, move toward increasingly effective therapies, and positively impact care and life pathways of patients and caregivers.

